# Improving oat yield and lodging resistance through nitrogen fertilization in the Alpine Qinghai-Tibet Plateau

**DOI:** 10.3389/fpls.2025.1684202

**Published:** 2026-01-05

**Authors:** Ruifang Zhang, Rui Wu, Wenhui Liu, Guoling Liang, Zeliang Ju

**Affiliations:** 1 Academy of Animal and Veterinary Sciences, Qinghai University, Qinghai Academy of Animal and Veterinary Sciences, Xining, Qinghai, China; 2 Key Laboratory of Qinghai Province Superior Forage Germplasm in the Oinghai-Tibet Plateau, Laboratory for Research and Utilization of Oinghai-Tibet Piateau Germplasm Resources, Xining, Qinghai, China

**Keywords:** oats (*Avena sativa*), nitrogen fertilizer, gradient, lodging, production

## Abstract

**Background:**

With the growing recognition of the nutritional, ecological, and economic importance of oats, global demand for oat production has been steadily increasing. Nitrogen (N) fertilization is a critical agronomic practice for enhancing crop productivity; however, excessive or inappropriate N application often results in lodging during oat growth/. Lodging not only compromises the high-yield potential of oats, but also reduces both grain and forage quality, representing a major constraint to achieving stable and sustainable production.

**Methods:**

To address this issue, two oat cultivars with contrasting lodging resistance—LENA (lodging-resistant) and QY2 (lodging-susceptible)—were evaluated under six N application rates (0, 60, 120, 180, 240, and 300 kg N·ha^-^¹). Measurements taken at the grain filling stage included agronomic traits, stem mechanical properties, biochemical composition, yield components, and lodging incidence.

**Results:**

Increasing N application significantly influenced PH, EH, CF_2_, CF_3_, and yield-related traits, all of which exhibited a nonlinear response that initially increased and subsequently declined at higher N levels. For LENA, the maximum fresh forage yield (61.78 t·ha^-^¹), hay yield (19.16 t·ha^-^¹), and seed yield (6.72 t·ha^-^¹) were obtained under the N3 treatment (180 kg N·ha^-^¹). Compared with the N0 control (0 kg N·ha^-^¹), these yields increased significantly by 19.80%, 21.56%, and 49.13% in 2018 (*P* < 0.05), and by 33.79%, 31.26%, and 17.12% in 2019 (*P* < 0.05), respectively. Simultaneously, the basal internode diameter, stem wall thickness of the 2nd and 3rd internodes, stem puncture strength, breaking strength, compressive strength, and lodging-related parameters (lodging rate and lodging index) exhibited an increasing trend with moderate N input. Principal determinants of stem lodging resistance were identified as PH, EH, HCG, basal internode length, and lignin content of the 3rd internode (LI_3_).

**Conclusion:**

This study demonstrates that nitrogen fertilization regulates lodging primarily by affecting the vertical elongation of basal internodes and promoting lignin biosynthesis. Under ecological conditions similar to those of the experimental site, the optimal N application rates for LENA and QY2 were 180 and 60 kg N·ha^-^¹, respectively. These N regimes effectively balance high yield potential with reduced lodging risk, and can be recommended for adoption in ecologically comparable oat-growing regions.

## 1 Introduction

Oats (*Avena sativa*) are a versatile annual grain and forage crop in the Poaceae family, valued for their cold tolerance, drought resistance, and adaptability to marginal soils and cool climates (Ahmad et al., 2020; Hilli and Kapoor, 2023). In the Qinghai-Tibet Plateau and adjacent regions, oats serve as premium green forage, silage, or hay, meeting the winter-spring nutritional demands of grazing livestock and alleviating pasture-livestock conflicts (Ahmad et al., 2014). Driven by the rising consumption of meat, eggs, and milk, the scale of cultivated oats has expanded, making yield improvement per unit area a central challenge for industrial development. Among agronomic practices, appropriate nitrogen fertilization is pivotal. An adequate nitrogen supply increases crop biomass, grain yield, and protein content (Duan et al., 2024). Excessive nitrogen prolongs vegetative growth and delays maturity, thereby increasing the risk of lodging (Hilli and Kapoor, 2023).

Lodging, which is the permanent tilting or bending of stems, impairs water and nutrient transport, alters canopy architecture, reduces light interception, and diminishes grain filling, thereby compromising the yield and quality (Wu and Ma, 2019). Predominantly occurring as stem or root lodging in forage grasses (Zhang et al., 2016; Ahmad et al., 2018), oats become particularly vulnerable during the transition to reproductive growth when spike weight increases and stem support weakens. Lodged plants create humid microenvironments that are conducive to pathogens and pre-harvest sprouting, further reducing harvest efficiency and increasing harvesting costs (Berry and Berry, 2015). In high-altitude pastoral systems, lodging is the primary barrier for achieving stable and high yields. Extreme weather, terrain, soil, and genetics influence lodging (Xu et al., 2017; Yu et al., 2017). Compared to altering environmental factors or breeding new varieties to modify crop genetic characteristics, adjusting cultivation practices is currently the most effective measure for achieving high and stable oat yields, and is also a key means of achieving high yield and quality. Research has shown that lodging resistance of stems is strongly linked to their morphology, physiology, and biomechanics (Liu et al., 2024). Studies have shown that plant height, center-of-gravity height, ear height, internode length, stem diameter, and stem wall thickness are closely associated with lodging resistance (Kelbert et al., 2004; Shen et al., 2018), and that nitrogen application modulates lodging by altering these traits. Recent studies have highlighted the importance of stem physicochemical traits as key factors for enhancing lodging tolerance. Cell wall rigidity is heavily dependent on the abundance of structural fibers (Caffall and Mohnen, 2009). Research conducted by Ma (2010) indicated the lignin content of stems in two wheat varieties with different lodging resistance and found that the lignin content of stems in the lodging-resistant variety was significantly higher than that in the lodging-susceptible variety at the milk stage. Other studies have indicated that decreased cellulose levels in soybean stems weaken their mechanical strength, resulting in an increased lodging risk (Liu et al., 2016). Early work by Mulder framed lodging as a failure point where the basal stem can no longer bear the mechanical load imposed above (Mulder, 1954) and this principle has since guided numerous biomechanical investigations into crop lodging resistance (Renaud et al., 2013). Yield reductions in oats caused by lodging range from approximately one-tenth to two-fifths, and in the most severe instances, can approach four-fifths of the potential harvest (Shah et al., 2017). Thus, in the alpine pastoral regions, lodging has emerged as the foremost constraint to achieving consistently high oat yields.

Direct optimization of agronomic practices, such as fertilization, represents a reliable approach to achieve both high yield and superior quality in oat production, in contrast to the slower processes of breeding lodging-resistant cultivars or elucidating lodging-related genetic mechanisms. However, significant challenges persist in reconciling the trade-off between high yield cultivation and lodging. In this study, we conducted a two-year (2018–2019) randomized complete-block field experiment in the hinterland of the Qinghai-Tibet Plateau. Two oat cultivars exhibiting contrasting lodging resistance were subjected to a gradient of nitrogen fertilizer treatments to investigate the regulatory effects of varying nitrogen levels on plant growth characteristics, lodging incidence, and yield formation. Our objective was to elucidate the interactive mechanisms underlying high-yield cultivation and lodging risk, thereby providing a theoretical foundation for the development of regionally optimized nitrogen management protocols.

## 2 Materials and methods

### 2.1 Study area

A two-year (2018–2019) field experiment was conducted in Ganhetan Town, Huangzhong District, Xining, Qinghai Province, China (101°33′22″ E, 36°30′55″ N), located in the core of the Qinghai-Tibet Plateau, with an average elevation of 2,628 m. The region exhibits a typical highland continental climate, characterized by an annual sunshine duration of 2 550–2–600 h and a mean annual temperature of 3.5°C–4.5°C, daily temperature range of 12°C–16°C, annual precipitation of 450–500 mm (concentrated from June to September), evaporation rate of 3–4 times the precipitation, and a frost-free period of approximately 90–120 days. The soil type in this region is chestnut-calcareous soil. Soil analyses showed that the site contained 58.2 g·kg^-^¹ organic matter, 3.1 g·kg^-^¹ total N, 54.3 mg·kg^-^¹ alkali-hydrolyzable N, 4.8 mg·kg^-^¹ available P, and 112.4 mg·kg^-^¹ available K, with pH ranging from 7.8 to 8.3.

### 2.2 Test materials

Oat cultivars LENA (Avena sativa cv. LENA), which exhibits high lodging resistance, and Qingyin No. 2 (hereafter QY2), a lodging-susceptible cultivar, were supplied by the Qinghai Academy of Animal Husbandry and Veterinary Sciences. Urea (46% N) served as the nitrogenous fertilizer.

### 2.3 Experimental design

This field experiment was arranged in a randomized block design and carried out from late April to late September in both 2018 and 2019, with six nitrogen application rates: (pure N): 0 kg·ha^-1^ (N0), 60 kg·ha^-1^ (N1), 120 kg·ha^-1^ (N2), 180 kg·ha^-1^ (N3), 240 kg·ha^-1^ (N4), and 300 kg·ha^-1^ (N5), with 12 treatments. Each plot had an area of 15 m² (3 m × 5 m) with three replicates, totaling 36 plots. For all treatments, nitrogen fertilizer was applied as a single basal dressing immediately before sowing, and was uniformly incorporated into the plow layer. The seeding rate was calculated based on the thousand-grain weight at a target plant density of 300,000 plants per acre, with rows spaced 20 cm apart and 1 m between plots. Superphosphate was applied as a basal fertilizer at 40 kg·ha^-1^ before sowing, and manual weeding was performed at the seedling, tillering, and jointing stages. The previous crop grown in the experimental area was barley.

### 2.4 Sampling and measurement

#### 2.4.1 Main agronomic traits

At the milk ripening stage, 15 uniformly vigorous plants per plot were randomly sampled, and the following traits were measured according to Liu et al. (2024): plant height (PH), height of the center of gravity (HCG), diameter of the second and third internodes (SD2 and SD3), wall thicknesses of the second and third internodes (WT2, WT3), lengths of the second and third internodes (SL2 and SL3), ear height (EH), and root fresh weight (RFW).

#### 2.4.2 Mechanical properties

At the milk-ripening stage, 15 uniform plants were randomly sampled per plot. A YYD-1 strength tester (Zhejiang Top Technology Co., Ltd.) was used to quantify the puncture strength (PS), breaking strength (BS), and compression strength (CS) across the second and third internodes.

PS was measured using a 1 mm² puncture probe. Stems stripped of sheaths were positioned in the device at 2 cm intervals, the probe was driven perpendicularly into the internode at a constant speed, and the peak force required to penetrate the epidermis was recorded.

BS was determined using a bending probe, and the maximum force causing stem fracture was recorded.

CS was assessed using a compression probe, which recorded the peak force required to deform the stem.

#### 2.4.3 Lodging

During the oat maturation period, the field lodging rate (FLR) was measured, and the lodging index (LI) was calculated using the following formula:

 FLR(%)=(lodging area)/(plot are) *100 (Peltonen-Sainio and Järvinen, 1995)

LI = (HCG × AFW)/BS2 (Wang et al., 2015).

BS2 denotes the breaking strength of the second stem internode.

#### 2.4.4 Stem physiological indicators

At the milk ripening stage, the second and third internodes were harvested, oven-fixed at 105°C for 30 min, dried to a constant weight at 65°C, milled through a 60-mesh sieve, and assayed for second- and third-internode crude fiber (CF2, CF3) and second- and third-internode lignin (LI2, LI3) following Liu et al (Liu et al., 2024).

#### 2.4.5 Yield measurement

Half of the milk-ripe samples were reserved to determine the fresh and dry forage yields. The remaining plants were harvested in their entirety at maturity to measure the seed yield.

### 2.5 Data analysis

Data were compiled and analyzed using Microsoft Excel 2019 and R 4.0.2. Analysis of variance (ANOVA) was performed, and mean differences were evaluated using Duncan’s multiple range test at *P* < 0.05. Pearson’s correlation analysis was conducted on the indicators related to plant lodging and lodging rate. We employed the PiecewiseSEM package to develop a piecewise structural equation model, elucidating the pathways and coefficients by which cultivar, nitrogen rate, and their interactions affect oat lodging. Using the plyr package, we applied the TOPSIS multi-criteria decision-making model to comprehensively evaluate lodging and yield, thereby identifying the optimal nitrogen rate for oat cultivation in the study region. Graphs were created using Origin 2024.

## 3 Result

### 3.1 Nitrogen fertilizer effects on agronomic traits

Between 2018 and 2019, plant height (PH), height of the center of gravity (HCG), diameters of the second and third internodes (SD2, SD3), wall thicknesses of the second and third internodes (WT2, WT3), lengths of the second and third internodes (SL2, SL3), ear height (EH), and root fresh weight (RFW) were measured (**Table 1**).

**Table 1:** Nitrogen fertilizer effects on oat agronomy.

Years	Varieties	F	PH	HCG	SD2	SD3	WT2	WT3	SL2	SL3	PFW	EH
2018	LENA	N0	73.85 ± 2.65c	31.80 ± 1.25c	2.76 ± 0.15c	2.92 ± 0.18d	0.55 ± 0.03b	0.54 ± 0.05c	3.13 ± 0.26a	6.27 ± 0.34a	0.89 ± 0.23a	59.99 ± 2.29c
		N1	84.72 ± 2.46b	36.96 ± 0.99b	2.90 ± 0.05bc	3.11 ± 0.07cd	0.56 ± 0.04b	0.55 ± 0.03bc	3.51 ± 0.35a	7.15 ± 0.66a	1.04 ± 0.19a	69.6 ± 2.62b
		N2	98.10 ± 3.28a	39.08 ± 1.77b	3.15 ± 0.14ab	3.45 ± 0.06bc	0.64 ± 0.04ab	0.57 ± 0.03abc	3.07 ± 0.08a	7.43 ± 0.29a	1.3 ± 0.15a	81.14 ± 3.14a
		N3	96.46 ± 0.81a	39.72 ± 1.09b	3.07 ± 0.11bc	3.42 ± 0.15bc	0.60 ± 0.03ab	0.59 ± 0.04abc	3.37 ± 0.25a	7.40 ± 0.31a	1.14 ± 0.05a	80.1 ± 1.17a
		N4	103.04 ± 2.41a	43.86 ± 1.55a	3.48 ± 0.08ab	3.88 ± 0.08a	0.67 ± 0.04a	0.67 ± 0.03ab	3.70 ± 0.26a	7.83 ± 0.83a	1.18 ± 0.25a	84.68 ± 2.74a
		N5	100.89 ± 2.72a	40.21 ± 0.94ab	3.49 ± 0.15a	3.73 ± 0.13ab	0.68 ± 0.02a	0.69 ± 0.04a	3.27 ± 0.27a	7.80 ± 0.58a	1.43 ± 0.23a	82.99 ± 2.19a
	QY2	N0	129.50 ± 1.04a	51.70 ± 0.56ab	2.61 ± 0.07bc	3.37 ± 0.11e	0.32 ± 0.01c	0.45 ± 0.01c	17.30 ± 0.43cd	23.89 ± 0.35b	0.4 ± 0.03c	114.3 ± 0.96ab
		N1	129.75 ± 1.80a	51.88 ± 0.62ab	2.70 ± 0.04b	4.05 ± 0.05b	0.53 ± 0.01a	0.46 ± 0.01c	16.58 ± 0.67d	23.82 ± 0.33b	0.65 ± 0.04b	117.09 ± 2a
		N2	126.00 ± 2.35ab	50.41 ± 0.84ab	2.52 ± 0.04bc	3.76 ± 0.13cd	0.45 ± 0.02b	0.45 ± 0.01c	18.86 ± 0.10b	23.82 ± 0.83b	0.67 ± 0.06b	109.33 ± 2.31b
		N3	123.75 ± 2.06bc	49.80 ± 1.20b	2.46 ± 0.07c	3.68 ± 0.11d	0.44 ± 0.01b	0.48 ± 0.02bc	18.43 ± 0.34bc	24.53 ± 0.46b	0.83 ± 0.04ab	109.07 ± 1.91b
		N4	120.00 ± 2.08c	51.54 ± 0.21ab	2.62 ± 0.10bc	4.00 ± 0.07bc	0.43 ± 0.02b	0.52 ± 0.03ab	20.62 ± 0.29a	25.72 ± 0.98b	1 ± 0.16a	102.9 ± 2.25c
		N5	120.50 ± 1.04bc	52.61 ± 0.33a	3.39 ± 0.09a	4.43 ± 0.04a	0.56 ± 0.01a	0.54 ± 0.01a	21.45 ± 0.69a	30.58 ± 0.87a	0.98 ± 0.03a	102.52 ± 1.19c
2019	LENA	N0	115.28 ± 0.43c	44.86 ± 2.09b	3.90 ± 0.11c	4.52 ± 0.05c	0.85 ± 0.02b	0.76 ± 0.01c	8.50 ± 0.21bc	14.05 ± 1.09a	1.43 ± 0.06a	98.19 ± 0.42d
		N1	128.81 ± 1.61a	52.94 ± 1.91a	4.54 ± 0.13b	5.04 ± 0.07bc	0.86 ± 0.01b	0.75 ± 0.02c	9.14 ± 0.34ab	15.75 ± 1.10a	1.27 ± 0.04b	110.94 ± 1.38a
		N2	127.41 ± 1.37ab	51.56 ± 1.04a	4.37 ± 0.14bc	4.80 ± 0.18bc	0.82 ± 0.13b	0.72 ± 0.01c	8.82 ± 0.21abc	16.33 ± 0.70a	0.99 ± 0.05c	108.44 ± 1.5a
		N3	127.04 ± 1.70ab	52.38 ± 2.37a	4.56 ± 0.15b	5.38 ± 0.27ab	0.88 ± 0.04b	0.76 ± 0.01c	9.19 ± 0.13a	15.88 ± 0.27a	0.82 ± 0.01d	107.71 ± 1.63ab
		N4	124.16 ± 1.59b	52.32 ± 1.64a	4.57 ± 0.53b	5.87 ± 0.30a	0.98 ± 0.05ab	0.81 ± 0.02b	9.35 ± 0.17a	16.91 ± 1.66a	1.07 ± 0.01c	103.8 ± 1.78bc
		N5	122.90 ± 2.90b	51.31 ± 1.23a	5.49 ± 0.28a	5.87 ± 0.23a	1.06 ± 0.08a	0.86 ± 0.01a	8.28 ± 0.11c	16.55 ± 0.16a	1.35 ± 0.01ab	100.34 ± 1.63cd
	QY2	N0	128.02 ± 0.77d	58.73 ± 1.14a	3.59 ± 0.11b	3.86 ± 0.15c	0.75 ± 0.01b	0.67 ± 0.01bc	17.71 ± 0.46c	25.35 ± 1.26d	1.34 ± 0.02a	108.91 ± 0.8cd
		N1	139.31 ± 2.06a	57.00 ± 1.06a	3.59 ± 0.07b	4.16 ± 0.07bc	0.75 ± 0.03b	0.66 ± 0.01c	19.03 ± 0.70c	25.70 ± 0.62d	1.26 ± 0.03ab	119.33 ± 2.04a
		N2	137.90 ± 0.76ab	56.85 ± 0.62a	3.56 ± 0.04b	4.15 ± 0.01bc	0.74 ± 0.02b	0.63 ± 0.01c	19.47 ± 0.45c	27.14 ± 0.31cd	1.05 ± 0.01cd	117.37 ± 0.61ab
		N3	133.63 ± 1.56bc	58.07 ± 2.64a	3.61 ± 0.07b	4.37 ± 0.15ab	0.72 ± 0.02b	0.66 ± 0.01c	15.64 ± 0.99d	28.71 ± 1.00bc	0.94 ± 0.07d	112.54 ± 1.86bc
		N4	128.96 ± 2.01cd	58.55 ± 1.46a	4.09 ± 0.29a	4.45 ± 0.09ab	0.77 ± 0.01b	0.71 ± 0.02ab	21.46 ± 0.79b	29.70 ± 0.55ab	1.15 ± 0.06bc	106.38 ± 2.19de
		N5	126.25 ± 1.80d	59.61 ± 1.32a	4.52 ± 0.20a	4.57 ± 0.15a	0.83 ± 0.01a	0.73 ± 0.01a	25.21 ± 0.38a	31.20 ± 0.31a	1.31 ± 0.02a	103.18 ± 1.56e

F, nitrogen fertilizer; PH, plant height (cm); HCG, height of center of gravity (cm); SD2 and SD3, diameters of the second and third stem internodes (mm); WT2 and WT3, wall thicknesses of the second and third stem internodes (mm); SL2 and SL3, lengths of the second and third stem internodes (cm); RFW, root fresh weight (g); EH, ear height (cm). The same as below. Letters denote significant differences at *P* < 0.05.

In 2018, as the nitrogen application rates increased, both oat varieties demonstrated a pattern of initial increases followed by subsequent decreases in PH and EH. LENA’s PH and EH were highest under N4, reaching 103.04 cm and 84.68 cm, respectively, reflecting significant increases of 39.53% and 41.16% compared to N0 (*P* < 0.05). However, no significant differences were observed among the N2, N3, and N5 treatments (*P* > 0.05). In contrast, QY2 exhibited the highest PH and EH under N1, but no significant differences were observed between N0 (PH: 129.75 cm *vs*. 129.50 cm; EH: 117.09 cm *vs*. 114.30 cm). SD2 of both LENA and QY2 increased with higher nitrogen application rates, peaking at N5. Significant increases of 26.45% and 29.89% were observed compared with N0. For LENA, SD3 showed a trend of first increasing and then decreasing with increasing nitrogen levels, reaching a maximum under N4, with a significant increase of 32.88% compared to that under N0. In contrast, SD3 of QY2 continued to increase with higher nitrogen application, peaking under N5, with a significant increase of 31.45% compared with N0. Nitrogen application had a significant effect on WT2 and WT3 for both the varieties. As the nitrogen rates increased, the wall thickness at these nodes increased, peaking under N5 and being significantly higher than that under N0. Nitrogen had a minimal effect on SL2 and SL3 of LENA, with no significant differences observed among the treatments. However, for QY2, SL2 and SL3 increased continuously with higher nitrogen application, peaking under N5, and was significantly greater than under N0. The HCG of LENA was significantly greater under nitrogen treatments than under N0, whereas no significant differences were found among the treatments for QY2. As nitrogen application increased, the RFW of LENA showed an increasing trend, peaking at 1.43 g under N5, although this was not significantly different from that under N0. For QY2, RFW first increased and then decreased with higher nitrogen application, reaching a peak of 1 g under N4, which was significantly greater than the 0.4 g observed under N0.

In 2019, as nitrogen application increased, the trends in PH and EH for both varieties were consistent with those observed in 2018, with both traits reaching their maximum values under N1 and being significantly higher than those under N0. Specifically, the PH and EH of LENA increased by 11.74% and 12.99%, respectively, compared to N0, while QY2 showed increases of 8.82% and 9.57%, respectively. As the nitrogen application rates increased, SD2 for both varieties followed the same pattern as in 2018, peaking under N5 and being significantly higher than that under N0. SD3 of LENA exhibited a trend of first increasing and then decreasing with higher nitrogen levels, reaching its maximum value under N4, where it increased significantly by 29.87% compared to the lowest value observed under N0. In contrast, SD3 of QY2 continued to increase with higher nitrogen application, peaking under N5, where it was significantly higher by 18.39% than that under N0. The effects of different nitrogen application treatments on WT2 and WT3 in both LENA and QY2 were found to be significant. As nitrogen application increased, the wall thickness of both stem nodes in both varieties thickened, reaching its maximum under N5 and being significantly higher than that under N0. With increasing nitrogen application, SL2 in LENA first increased and then decreased, reaching its maximum value under N4, which was significantly higher than that under N0 (9.35 cm *vs*. 8.50 cm). No significant differences were observed in SL3 among the nitrogen treatments (*P* > 0.05). For QY2, SL2, and SL3, the values increased continuously with higher nitrogen application, reaching their maximum values under N5 and being significantly higher than those under N0. HCG for LENA was significantly higher under all nitrogen treatments than under N0, with no significant differences observed among the nitrogen treatments (*P* > 0.05). For QY2, no significant differences were observed between the treatments (*P* > 0.05). The RFW of both LENA and QY2 was greatest under N0, with values of 1.43 g and 1.34 g, respectively.

### 3.2 Nitrogen fertilizer effects on stem mechanical properties

**Figure 1:**
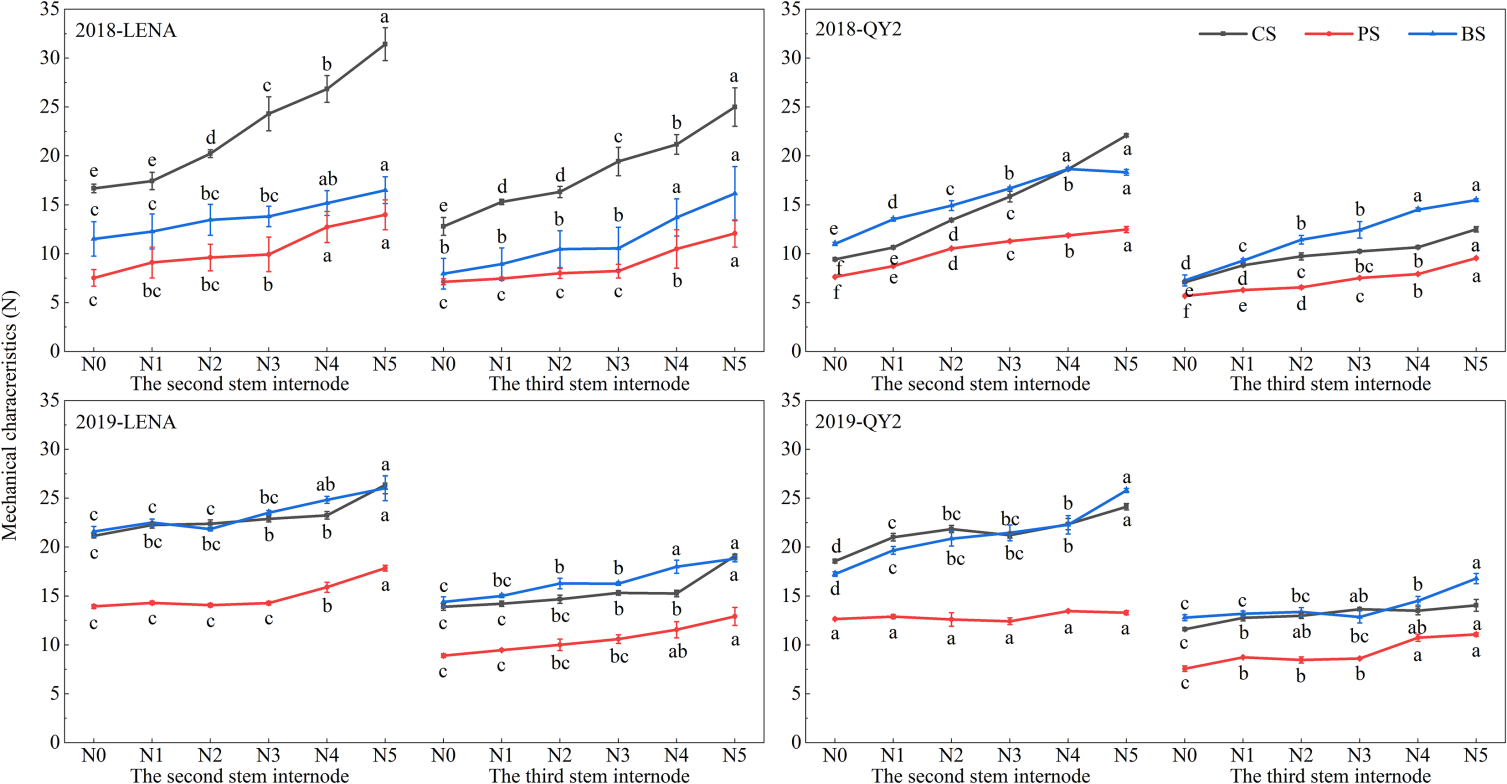
Effects of nitrogen application rates on the mechanical properties of two oat cultivars grown in 2018 and 2019. Letters denote significant differences at *P* < 0.05.

**Figure 1** illustrates that puncture strength (PS), breaking strength (BS) and compression strength (CS) of both second- and third-internodes increased with higher nitrogen application. At every nitrogen level, the resistant cultivar LENA exhibited greater PS, BS, and CS than the susceptible QY2. Moreover, for both cultivars and across all treatments, the values recorded at the second internode exceeded those at the third.

### 3.3 Nitrogen fertilizer effects on stem physiological characteristics

**Figure 2:**
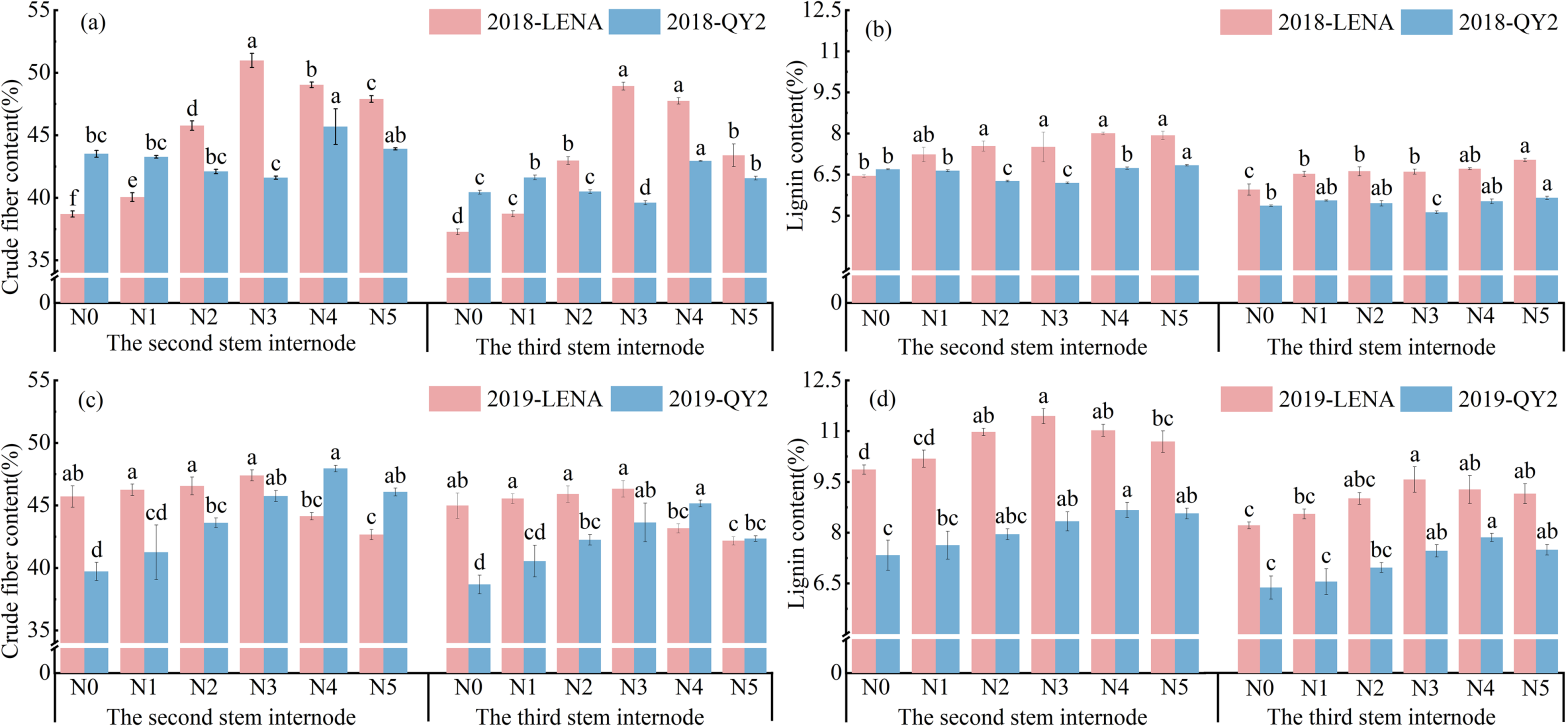
Effects of nitrogen application rates on crude fiber content **(a, c)** and lignin content **(b, d)** of two oat cultivars grown in 2018 and 2019. Letters denote significant differences at *P* < 0.05.

As nitrogen application rates increased, crude fiber content at the second (CF2) and third internodes (CF3) of both LENA and QY2 initially increased, then decreased, with CF2 consistently higher than CF3 (**Figures 2a, c**). For LENA, both CF2 and CF3 reached their maxima under N3 treatment. In 2018, CF2 and CF3 increased significantly by 31.7% and 31.3%, respectively, relative to the N0 treatment (*P* < 0.05), whereas in 2019, no significant differences were observed compared to N0. For QY2, CF2, and CF3 peaked under N4 treatment. In 2018, CF2 and CF3 increased by 45.7% and 43.0%, respectively, compared with N0. In 2019, CF2 and CF3 increased by 15.2% and 16.7%, respectively, compared to the N0 treatment.

Basal lignin concentration was higher in the lodging-tolerant cultivar LENA than in the lodging-prone QY2 (**Figures 2b, d**). Under the same nitrogen application rate, the lignin and crude fiber contents at the second internode were greater than those at the third internode for both cultivars. In 2018, as nitrogen application rates increased, the lodging-resistant variety LENA exhibited a trend of first increasing, then decreasing in LI2, which peaked at 8.00% under the N4 treatment, which was significantly higher than the 6.45% observed under N0. LI3 in LENA showed a continuous increase, reaching 7.03% under the N5 treatment, which was significantly higher than 5.95% under N0. In contrast, LI2 and LI3 of the susceptible QY2 continuously increased with increasing nitrogen application, reaching 6.84% and 5.45%, respectively, under N5, which was significantly higher than that under N0. In 2019, both LI2 and LI3 of LENA and QY2 followed a trend of first increasing, then decreasing, with increasing nitrogen application. LENA reached its maximum value under N3 treatment, with LI2 and LI3 increasing by 16.1% and 16.5%, respectively, compared to N0. QY2 reached its maximum under the N4 treatment, with increases of 18.2% and 23.2%, respectively, compared to N0.

### 3.4 Nitrogen fertilizer effects on stem lodging characteristics

**Figure 3:**
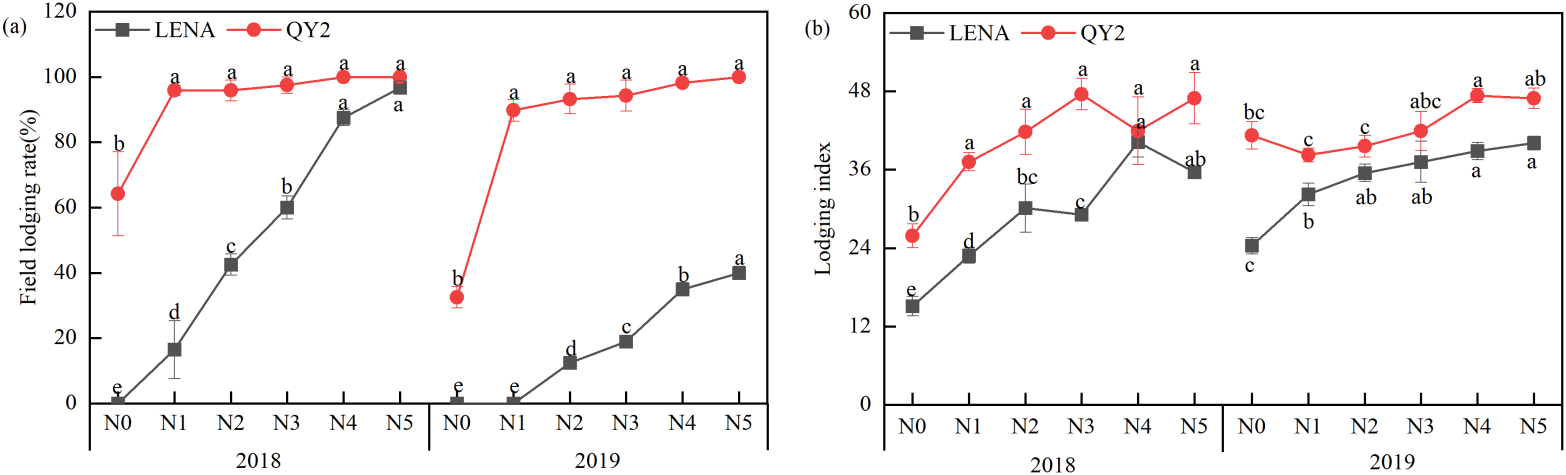
Effects of nitrogen application rates on lodging incidence **(a)** and lodging index **(b)** of two oat cultivars grown in 2018 and 2019. Letters denote significant differences at *P* < 0.05.

Different nitrogen application rates significantly influenced oat lodging incidence and lodging index (**Figures 3a, b**). With increasing nitrogen, both metrics increased for the susceptible cultivar QY2 and the tolerant cultivar LENA, with QY2 consistently exhibiting higher values. Across both seasons, QY2’s lodging incidence under all nitrogen treatments exceeded that under N0 (*P* < 0.05). For LENA, lodging incidence in 2018 under nitrogen treatments was greater than under N0 (*P* < 0.05), whereas in 2019, no significant difference was observed between N0 and N1, although both remained lower than under higher nitrogen rates. Except for QY2 in 2019, lodging index values were significantly elevated under nitrogen treatments compared with N0 (*P* < 0.05).

### 3.5 Effect of nitrogen fertilizer on yield

**Figure 4:**
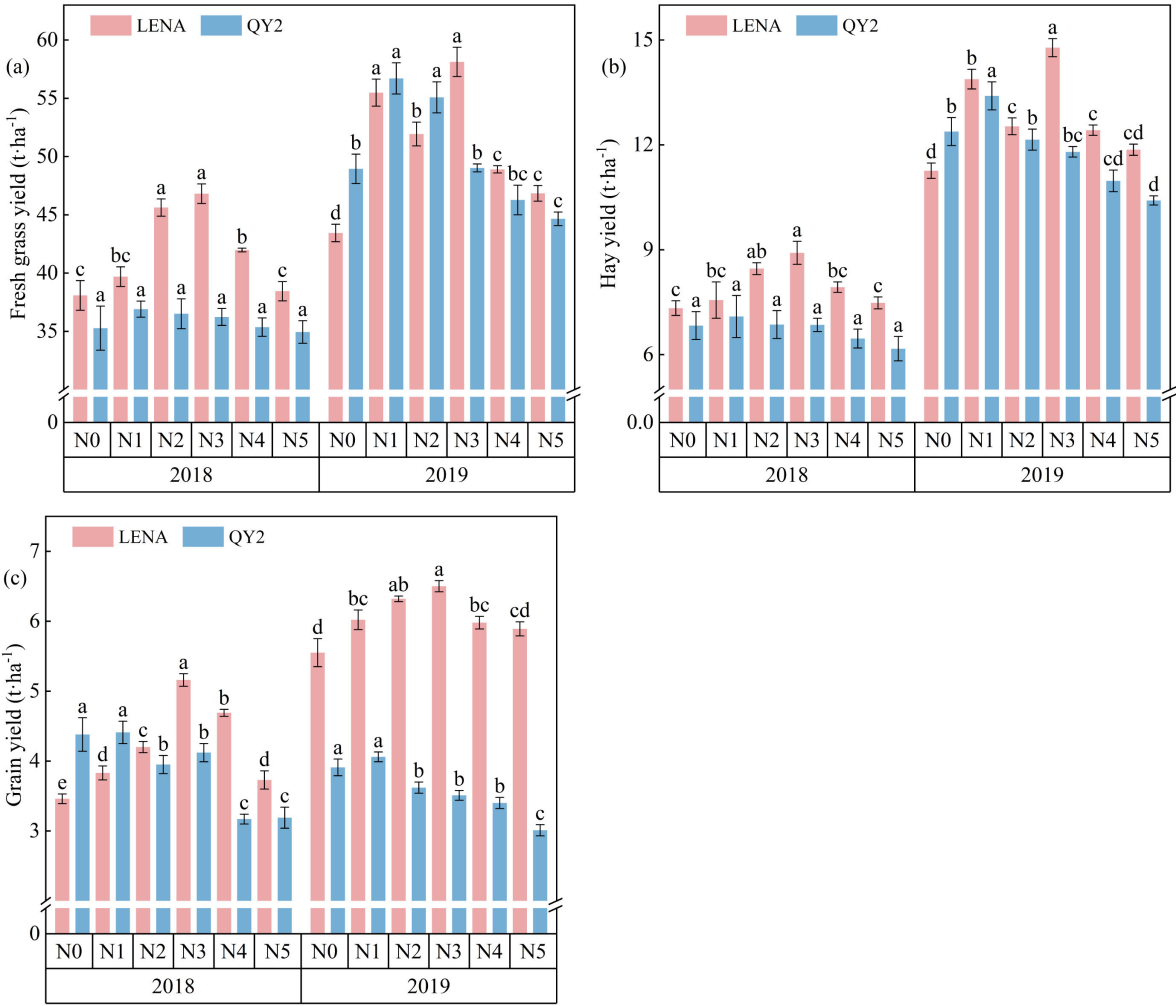
The influence of nitrogen fertilizer on yield is represented by **(a-c)**, which stand for fresh grass yield, dry grass yield, and grain yield, respectively. Different letters indicate significant differences at P < 0.05.

Fresh forage yield, dry forage yield and seed yield of both cultivars initially increased with rising nitrogen application rates before declining (**Figure 4**). In 2018, LENA’s yields peaked under the N3 treatment, rising by 19.8%, 21.6% and 49.1% for fresh forage, dry forage and seed, respectively, relative to N0 (*P* < 0.05). For QY2, all three yields attained maxima under N1 (36.9, 7.09, and 4.41 t·ha^-1^, respectively), although these differences were not significant versus N0. In 2019, LENA again peaked under N3, with fresh forage, dry forage, and seed yields increasing by 33.8%, 31.3%, and 17.1% over N0 (*P* < 0.05). QY2’s fresh and dry forage yields peaked under N1, increasing by 15.9% and 8.2%, respectively, while its seed yield (4.06 t·ha^-1^) did not differ significantly from that under N0.

Results from the two-year experiment showed that the lodging-resistant cultivar LENA and the susceptible cultivar QY2 achieved the highest forage and grain yields under the N3 (180 kg·ha^-^¹) and N1 (60 kg·ha^-^¹) treatments, respectively.

### 3.6 Analysis of variance

**Table 2** presents the effects of growing year, variety, nitrogen application rate, and their interactions on agronomic traits, mechanical properties, biochemical composition, lodging, and yield of oats. The nitrogen application rate had a highly significant effect on all indicators (*P* < 0.01), and variety and growing year had significant effects on most indicators. However, there was no significant difference in BS2 among varieties (*P* > 0.05), and the growing year also had no significant effect on CF2 and CS3.

**Table 2:** Analysis of variance.

Sources of variation	CF2	LI2	CF3	LI3	CS2	CS3	PS2	PS3	BS2	BS3	FGY	HY		GY
V	40.215**	351.242**	91.93**	343.142**	651.44**	1165.323**	39.039**	78.833**	0.82	16.033**	29.703**	74.796**		36.952**
F	24.235**	11.392**	17.356**	9.836**	239.776**	126.893**	45.209**	53.366**	59.103**	51.074**	18.472**	17.235**		4.909**
Y	1.931	652.596**	26.598**	643.807**	339.139**	1.043	388.124**	113.332**	997.906**	197.102**	371.59**	1581.802**		90.888**
V×F	6.304**	3.051*	4.991**	1.79	2.905*	23.334**	5.993**	0.94	4.156**	0.182	6.519**	5.86**		12.104**
V×Y	1.484	86.045**	1.176	19.728**	294.497**	379.218**	34.898**	0.621	64.937**	32.721**	18.736**	1.402		218.539**
F×Y	8.07**	2.997*	16.102**	5.769**	69.87**	23.428**	9.909**	0.719	1.521	6.173**	3.716**	5.801**		2.091
V×F×Y	50.97**	1.965	49.665**	1.853	1.255	6.851**	1.07	1.416	1.587	1.867	2.226	6.363**		2.735*
Sources of variation	PH	SD2	SD3	WT2	WT3	SL2	SL3	HCG	PFW	EH	FLR		LC	LI
V	687.375**	117.051**	21.059**	148.539**	132.465**	5409.508**	2356.365**	313.394**	19.838**	651.917**	1135.04**		144.295**	104.001**
F	15.699**	29.256**	26.409**	13.777**	10.5**	29.81**	15.044**	5.751**	4.538**	12.898**	124.062**		6.326**	27.609**
Y	643.531**	555.851**	374.341**	551.638**	392.349**	319.616**	323.772**	287.124**	21.746**	396.993**	156.669**		35.134**	18.822**
V×F	24.39**	2.185	2.872*	2.759*	3.352**	33.009**	6.566**	6.754**	0.877	23.617**	24.393**		8.171**	1.708
V×Y	245.548**	8.706**	160.968**	0.414	0.824	168.241**	94.31**	23.618**	23.339**	296.13**	61.302**		94.921**	3.496
F×Y	6.19**	1.748	1.3	2.364*	1.487	5.231**	1.237	0.592	8.497**	7.005**	2.981*		5.333**	3.422**
V×F×Y	10.339**	2.964*	2.137	2.46*	1.594	7.693**	0.78	1.28	0.403	10.815**	16.878**		5.645**	3.225*

* and ** denote significant differences at *P* 0.05 and *P* 0.01, respectively. Y, growth year; V, oat variety; F, fertilization.3.2 Nitrogen fertilizer effects on stem mechanical properties

Further analysis of the effects of interactions revealed that the interaction between variety and nitrogen application rate had a highly significant impact on indicators such as crude fiber content, forage yield, seed yield, PH, HCG, internode length, and lodging rate (*P* < 0.01). The interaction between variety and growing year also had a highly significant effect on indicators such as lignin content, compressive strength, tensile strength, fresh forage yield, seed yield, PH, HCG, stem diameter, internode length, FRW, and lodging rate. Additionally, the interaction between nitrogen application rate and growing year had a highly significant effect on crude fiber content, stem node compressive strength, forage yield, PH, and indicators related to the ear and root regions of the plants. The interaction among variety, nitrogen application rate, and growing year had a highly significant effect on crude fiber content, forage yield, PH, EH, and lodging rate.

### 3.7 Main factors affecting the lodging rate of oats

**Figure 5:**
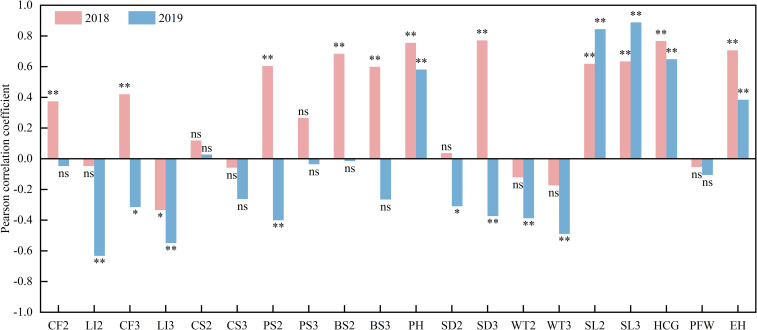
Correlation analysis of field lodging rate with stem morphology indicators, mechanical features and physiological indicators in 2018 and 2019. (* and ** denote significant differences at *P* < 0.05 and *P* < 0.01, respectively. ns, represents not significant.).

Key factors influencing oat lodging rates over a two-year period were identified (**Figure 5**). The analysis results showed that in 2018, the crude fiber content, second internode puncture strength, second and third internode break strengths, PH, SD3, SL2, SL3, HCG, and EH were extremely significantly positively correlated with the lodging rate (*P* < 0.01). whereas LI3 was significantly negatively correlated with the lodging rate (*P* < 0.05). In 2019, PH, SL2 and SL3, HCG, and EH were extremely significantly positively correlated with lodging rate; LI2 and LI3, second stem node puncture strength, SD3, WT2 and WT3, and lodging rate were extremely significantly negatively correlated, while CF3 and SD2 were significantly negatively correlated with lodging rate.

**Figure 6:**
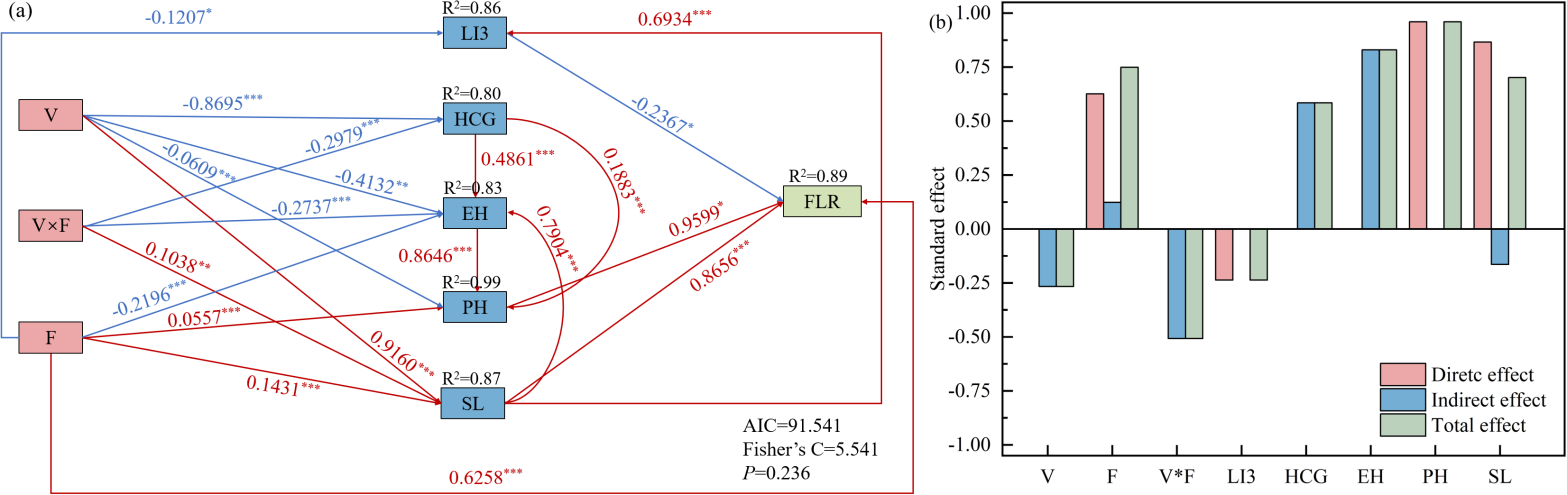
Structural equation modeling was used to evaluate the effects of cultivar and nitrogen application rate on oat lodging **(a)**. Direct, indirect and total effects of each variable **(b)**. Solid pink and blue arrows denote significant positive and negative relationships, respectively (P < 0.05). The significance levels are indicated as *P < 0.05, **P < 0.01, and ***P < 0.001.

A structural equation model was constructed using significant factors such as PH, HCG, EH, internode length at the base (second and third stem nodes), and LI3, which showed consistent correlations with the lodging rate over a two-year period. The model was used to investigate the effects of variety, nitrogen application rate, the interaction between variety and nitrogen application rate, and related indicators on lodging rate (**Figure 6a**). The model fit was good, *P* = 0.236 > 0.05, Fisher’s C = 5.541, and AIC (Akaike information criterion) = 91.541, allowing for further analysis. The model results showed that the nitrogen application rate had a highly significant positive direct effect on the lodging rate, with a path coefficient of 0.6258. LI3 had a direct and extremely significant negative effect on the lodging rate, with a path coefficient of -0.2367. PH and basal internode length had direct and extremely significant positive effects on the lodging rate, with path coefficients of 0.9599 and 0.8656, respectively. Additionally, variety, nitrogen application rate, and their interaction indirectly influence oat lodging rate by affecting LI3, HCG, EH, PH, and basal internode length, with total effect values of -0.2367, 0.5842, 0.8299, 0.9599, and 0.7015, respectively (**Figure 6b**). In summary, variety and nitrogen application rate primarily influenced lodging rate by affecting the length of the basal internodes (second and third stem nodes), PH, and LI3.

### 3.8 Comprehensive evaluation

**Figure 7:**
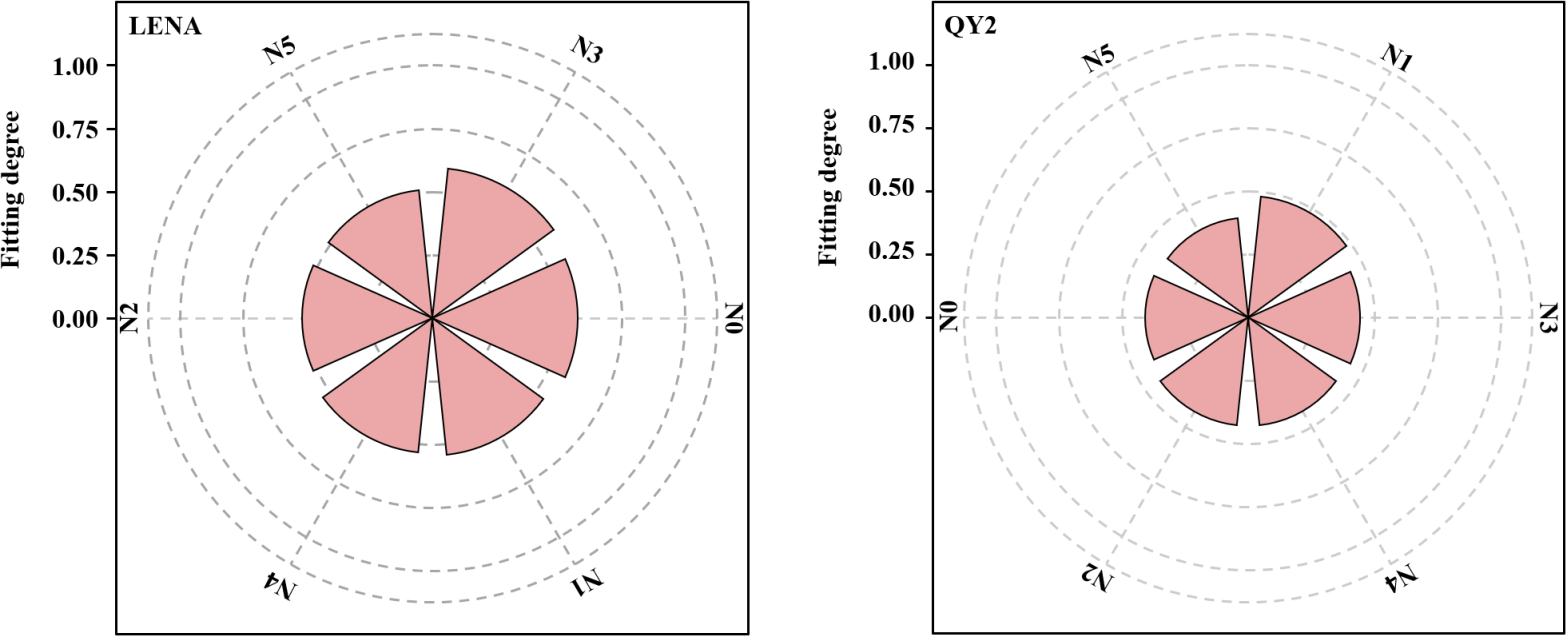
Comprehensive evaluation of yield and lodging traits of the two varieties.

A comprehensive evaluation based on indicators, such as lodging and yield, revealed that the optimal nitrogen application rates differed between the two varieties (**Figure 7**). LENA exhibited the highest correlation coefficient (0.61) under the N3 treatment and the lowest (0.50) under the N5 treatment. QY2 showed the highest correlation coefficient (0.48) under the N1 treatment and the lowest (0.39) under the N5 treatment. Therefore, LENA and QY2 maintained both high production performance and low lodging rates under N3 and N1 treatments, respectively, making them ideal nitrogen application rates for high-altitude cold regions.

## 4 Discussion

Nitrogen application has a significant impact on crop lodging rates. As nitrogen fertilizer application rates increased, lodging rates showed an upward trend. When nitrogen fertilizer application reached 180 kg·ha^-1^, rice (*Oryza sativa* L.) lodging rates reached 50%, which was significantly higher than that of the control (Dhakal et al., 2021; Wu et al., 2023). Similar results were obtained in this study, with both the lodging index and lodging rate increasing as nitrogen application rates increased, indicating that, within a certain range, increased nitrogen application leads to weakened lodging resistance. This may be because excessive nitrogen application promotes excessive stem elongation in oats, thins cell walls, and increases PH and HCG, thereby increasing the risk of lodging. In this experiment, regardless of nitrogen application rate, the lodging rate of the QY2 variety was consistently higher than that of LENA, indicating that under natural conditions, the occurrence of lodging is closely related to the genetic characteristics of the variety itself (Liu et al., 2024).

Numerous factors influence lodging resistance, and key morphological indicators for assessing lodging resistance include PH, HCG, length of the basal internode, stem diameter, and wall thickness (Liu et al., 2024). Therefore, this study quantified the morphological traits of the second and third internodes, together with PH and HCG. The nitrogen application rate exerted a highly significant influence on these variables (*P* < 0.01), and each variable was strongly correlated with lodging incidence. Some studies have indicated that shorter plants and compressed lower internodes enhance standability (Rajala et al., 2002; Tripathi et al., 2003). Additionally, studies have revealed that as the center of gravity increases, the lodging risk increases (Khan et al., 2017). Correlation analysis indicated that PH, internode length, and HCG were significantly positively correlated with lodging rate, consistent with previous findings. Therefore, in breeding processes, varieties with relatively shorter plant heights should be selected to assess their potential lodging resistance, and appropriate plant heights should be maintained to enhance yield. As nitrogen rates increased, the PH of both varieties initially increased and then decreased, indicating that within a certain range, nitrogen application can supply the nutrients required for oat growth and development, thereby promoting growth; however, excessive nitrogen application inhibits growth (Zhai et al., 2022a). Previous studies have shown that crop resistance to lodging is primarily attributed to increased stem thickness, which enhances the resistance of the stem to breakage (Hirano et al., 2014). However, our findings diverge from those of previous reports. Although the stem thickness of the lodging-resistant variety was greater than that of the lodging-prone variety, the correlation analysis indicated that the lodging rate in 2018 was not significantly correlated with stem thickness or stem wall thickness. This may be because the region experienced extreme weather on July 23, 2018, with a daily rainfall of 30.4 mm (meteorological data were sourced from era5: https://cds.climate.copernicus.eu). At this time, oats were in the late flowering stage or early grain-filling stage, with plants transitioning from vegetative to reproductive growth, and heavier spikes. Widespread stem lodging occurred under the influence of heavy rainfall. Meanwhile, no extreme weather occurred during the 2019 growing season. The stem diameter and wall thickness of the second and third stem nodes increased with increasing nitrogen application rates, and these two traits were negatively correlated with lodging rate. This indicates that under stable growing conditions (without heavy rainfall or strong winds), appropriate nitrogen fertilization can enhance stem growth, thereby improving lodging resistance to some extent (Zhai et al., 2022b). Therefore, these indicators can serve as a basis for evaluating crop lodging resistance, but they should be considered in conjunction with actual conditions, such as the climate of the planting site. Related studies have shown that the higher the plant height, the higher the center of gravity, and the longer the basal internode length, the more likely the crop is to lodge under wind or other external forces (Cruz et al., 2001). Conversely, the larger the basal internode diameter and the thicker the stem wall, the lower the lodging rate and the stronger the lodging resistance (Islam et al., 2007). Based on the above findings, a comparison of stem morphological indicators between the LENA and QY2 varieties revealed that the lodging-resistant variety LENA had shorter second and third stem internodes, lower plant height, and lower center of gravity height, but thicker stems and walls. This result is consistent with previous studies, further confirming the importance of stem morphological indicators in assessing crop lodging resistance.

Lignin, a principal constituent of plant cell walls, substantially enhances stem mechanical rigidity and confers resistance to lodging (Lewis and Yamamoto, 1990; Turner and Somerville, 1997). Research indicates that lignin significantly enhances stem compressive strength and fracture resistance by promoting lignification (Baucher et al., 1998). Stem mechanical strength is typically evaluated using indicators, such as puncture strength, fracture strength, and compressive strength, which are closely related to the content of lignin and cellulose in the stem (Ookawa et al., 2010; Sun et al., 2022). In studies of crop lodging resistance mechanisms, lignin content has been confirmed as an important indicator for assessing lodging resistance. Xia et al. found in corn (*Zea mays*) that varieties with poor lodging resistance had lower lignin accumulation than those with strong lodging resistance (Xia et al., 2013). Additionally, multiple studies have shown that varieties with a higher lignin content typically exhibit stronger stem bending resistance (Berry et al., 2003; Tripathi et al., 2003; Wang et al., 2014). In this study, the lignin content in the basal internodes of the lodging-resistant variety LENA was higher than that of the lodging-susceptible variety QY2, further supporting this conclusion. The effect of nitrogen fertilizer application on stem mechanical properties primarily manifests through its regulatory role in lignin synthesis. Studies have shown that under moderate nitrogen application rates (192 kg·ha^-1^), wheat stem lignin content reaches its peak, significantly enhancing lodging resistance (Murozuka et al., 2014). Appropriate nitrogen fertilizer application can activate the activity of key enzymes involved in lignin synthesis, thereby increasing lignin content in crop stems and ultimately enhancing resistance to lodging (Ahmad et al., 2023). Moderate nitrogen application can enhance the mechanical strength of plant stems (Zhao et al., 2023). In this study, the puncture strength, fracture strength, and compressive strength of both varieties increased with increasing nitrogen application rates. However, the lodging rate also exhibited a similar trend, suggesting that mechanical strength may not be the primary factor influencing lodging. The correlation analysis results of this study indicate that PH, HCG, EH, basal internode length, and LI3 are significantly correlated with lodging rate, while mechanical strength shows no obvious relationship with it. This partially explains that mechanical strength is only a secondary influencing factor of lodging, and it only affects lodging to a certain extent.

In high-altitude, cold environments, oat lodging critically constrains both yield potential and consistent production. Nitrogen fertilizer is an important crop management measure, and appropriate nitrogen fertilizer application can effectively promote oat growth, maintain lodging rates within an acceptable range, and maximize the crop’s production potential. Research indicates that nitrogen fertilizer application rates significantly influence oat yield, with appropriate nitrogen fertilizer application enhancing both forage yield and grain quality (Tobiasz-Salach et al., 2023). However, excessive nitrogen application not only reduces the number of grains per spike and spike weight but also increases plant height, which in turn raises lodging susceptibility and results in yield penalties (Kolmanič et al., 2022). Studies report that once the nitrogen saturation point is exceeded, further inputs fail to boost per-unit-area yields (Zhao et al., 2020). The main findings of this study can be summarized as follows: the lodging-resistant cultivar LENA maintained both high productivity and a low lodging rate under the N3 rate (180 kg·ha^-^¹); therefore, N3 is still recommended as the optimum nitrogen level for this cultivar. In contrast, the lodging-susceptible cultivar QY2 exhibited a markedly different response pattern. Across both experimental years, the highest forage and seed yields were observed under the N1 treatment (60 kg·ha^-^¹). Although these yield improvements were not statistically significant compared with N0, the economic analysis revealed that N1 increased net profit by 286.78–1,334.33 CNY·ha^-^¹ relative to N0 (**Supplementary Table S1**). Taken together, these results suggest that for QY2, the N1 treatment provides a balanced trade-off among productivity, lodging resistance, and economic return, and can therefore be recommended as the optimal nitrogen regime for this cultivar.

## 5 Conclusion

Nitrogen fertilizer exerts a significant influence on the growth, lodging incidence, and yield of oats (*P* < 0.05). The rational application of nitrogen fertilizer modulates the lodging behavior of oats by regulating their morphological traits, encompassing PH, HCG, EH, and basal internode length. Additionally, it impacts stem mechanical strength by modulating lignin synthesis in the stem, thereby influencing the lodging rate. Indicators such as PH, HCG, EH, internode length of the basal (second and third stem nodes) segments, and LI3 are closely associated with lodging resistance and can be employed to evaluate the lodging resistance performance of oats. Overall, LENA and QY2 achieved both high productivity and low lodging under 180 kg·ha^-^¹ (N3) and 60 kg·ha^-^¹ (N1), respectively. These rates represent the optimal nitrogen regimes for oat production in the study area and can be recommended for similar agro-ecological regions.

## Data Availability

The original contributions presented in the study are included in the article/supplementary material. Further inquiries can be directed to the corresponding author.
