# Supplementary material for: Improving oat yield and lodging resistance through nitrogen fertilization in the Alpine Qinghai-Tibet Plateau
Source: Front Plant Sci. 2026 Jan 5;16:1684202. doi: 10.3389/fpls.2025.1684202 (PMC12812906; doi:10.3389/fpls.2025.1684202)
Supplement: Supplementary file 1 [file DataSheet1.pdf]

Supplementary Material

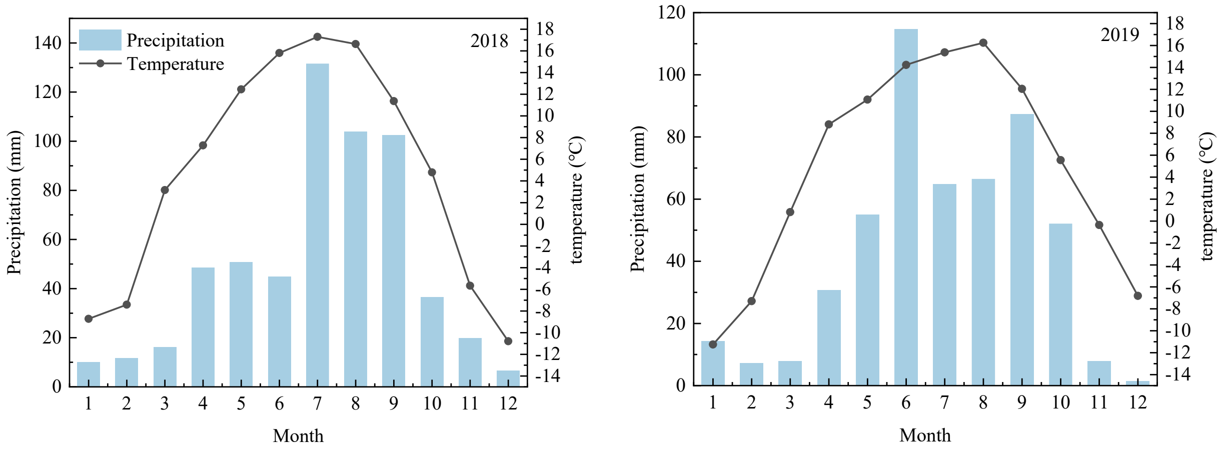

Figure S1 Monthly mean temperature and monthly total precipitation in the study area in 2018 and 2019.

Table S1 Economic Benefit Calculation of Oats under Different Nitrogen Fertilization Treatments (2018–2019)

Year	Treatment	Hay Yield (t/hm²)	Hay Price (CNY/t)	Hay Output Value (CNY/hm²)	Seed Yield (t/hm²)	Seed Price (CNY/kg)	Seed Output Value (CNY/hm²)	Total Output Value (CNY/hm²)	Nitrogen Application Cost (CNY/hm²)	Economic Benefit (CNY/hm²)	Input-Output Ratio (Total Output Value / Fertilizer Cost)	Benefit Difference vs. N0 (CNY/hm²)
2018	N0	6.17	2419.93	≈14931.97	4.41	6	26460	≈41391.97	0	≈41391.97	-	-
2018	N1	6.46	2419.93	≈15632.75	4.38	6	26280	≈41912.75	234	≈41678.75	≈179.11:1	≈+286.78
2019	N0	12.38	2419.93	≈29958.73	4.06	6	24360	≈54318.73	0	≈54318.73	-	-
2019	N1	13.4	2419.93	≈32427.06	3.91	6	24360	≈55887.06	234	≈55653.06	≈238.83:1	≈+1334.33
